# [Corrigendum] CDCA5 promotes the progression of prostate cancer by affecting the ERK signalling pathway

**DOI:** 10.3892/or.2023.8602

**Published:** 2023-07-17

**Authors:** Junpeng Ji, Tianyu Shen, Yang Li, Yixi Liu, Zhiqun Shang, Yuanjie Niu

Oncol Rep 45: 921–932, 2021; DOI: 10.3892/or.2021.7920

Subsequently to the publication of the above paper, an interested reader drew to the authors' attention that a pair of the data panels showing the results of Transwell invasion assays (specifically, the ‘C4-2 / shCON’ and the PC-3 / CON panels) featured in Fig. 3F on p. 927 contained overlapping sections, such that data that were intended to show the results from differently performed experiments appeared to have been derived from the same original source.

The authors were able to re-examine their original data files, and realized that this figure had been inadverently assembled incorrectly. The revised version of Fig. 3, containing the correct representative image of the PC-3shCON group, is shown on the next page. The authors also noted that the statistics in Fig. 3G remained correct, and did not require correction on account of the error made in assembling this figure. Similarly, note that the correction made to this figure does not affect the overall conclusions reported in the paper. The authors are grateful to the Editor of *Oncology Reports* for allowing them the opportunity to publish this Corrigendum, and all the authors agree with its publication. They also apologize to the readership for any inconvenience caused.

## Figures and Tables

**Figure 3.**
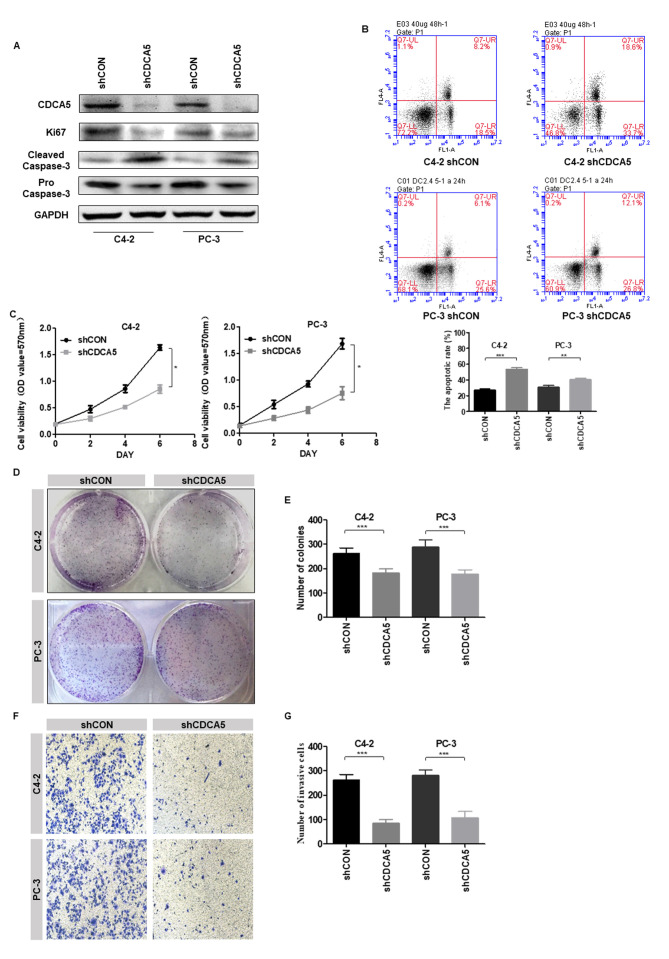
Knockdown of CDCA5 inhibits PCa cell proliferation and invasion *in vitro*. (A) Western blotting was used to detect the expression levels of CDCA5, Ki67 and cleaved/pro caspase-3 after CDCA5 knockdown in C4-2 and PC-3 cells. GAPDH was used as an internal control. (B) Flow cytometry was used to detect apoptosis in C4-2 and PC-3 cells after CDCA5 knockdown. The apoptotic rate (early apoptosis + late apoptosis) of C4-2 and PC-3 after knockdown CDCA5 was counted and plotted on a graph (**P<0.01 and ***P<0.001). The UL region indicates cell necrosis, the LL region indicates cell survival, the LR region indicates early cell apoptosis, and the UR region indicates late cell apoptosis. The LR region + UR region indicate total apoptosis. (C) An MTT assay was used to detect C4-2 and PC-3 cell growth after CDCA5 was knocked down. The absorbance value at a wavelength of 570 nm was detected (*P<0.05). (D) A colony formation assay was used to detect C4-2 and PC-3 cell growth after CDCA5 knockdown. (E) The number of colonies from D was counted and plotted (***P<0.001). (F) Transwell invasion assays detected the invasiveness of the prostate cancer cells after knockdown of CDCA5. (G) The number of invaded cells in F was counted and plotted on a graph (***P<0.001). CDCA5, cell division cycle-associated 5; PCa, prostate cancer; sh, short hairpin; CON, control; UL, upper left; LL, lower left; LR, lower right; UR, upper right..

